# Imperfect Breathing

**Published:** 1884-03

**Authors:** 


IMPERFECT BREATHING. IMPURE BLOOD.

Surely it will not be difficult to remember these two things. We thus can plainly see how it is that persons who sit a great deal become consumptive; and any one may apply it to himself in the various occupations of life, without any further specifications as to this branch of the causes of Consumption.

